# (*Z*)-*N*,*N*-Dimethyl-2-[phen­yl(pyridin-2-yl)methyl­idene]hydrazinecarbothio­amide

**DOI:** 10.1107/S1600536811045739

**Published:** 2011-11-05

**Authors:** K. Jayakumar, M. Sithambaresan, M. R. Prathapachandra Kurup

**Affiliations:** a Department of Applied Chemistry, Cochin University of Science and Technology, Kochi 682 022, India; b Department of Chemistry, Faculty of Science, Eastern University, Sri Lanka, Chenkalady, Sri Lanka

## Abstract

The title compound, C_15_H_16_N_4_S, exists in the *Z* conformation with the thionyl S atom lying *cis* to the azomethine N atom. The shortening of the N—N distance [1.3697 (17) Å] is due to extensive delocalization with the pyridine ring. The hydrazine–carbothio­amide unit is almost planar, with a maximum deviation of 0.013 (2) Å for the amide N atom. The stability of this conformation is favoured by the formation of an intra­molecular N—H⋯N hydrogen bond. The packing of the mol­ecules involves no classical inter­molecular hydrogen-bonding inter­actions; however, a C—H⋯π inter­action occurs.

## Related literature

For abackground to hydrazinecarbothio­amide and its derivatives, see: Beraldo & Gambino (2004 ▶). For the synthesis, see: Joseph *et al.* (2006 ▶). For related structures of hydrazinecarbothio­amides, see: Philip *et al.* (2006 ▶); Arumugam *et al.* (2011 ▶). For related structures, see: Seena *et al.* (2008 ▶); Usman *et al.* (2002 ▶); Huheey *et al.* (1993 ▶); Joseph *et al.* (2004 ▶).
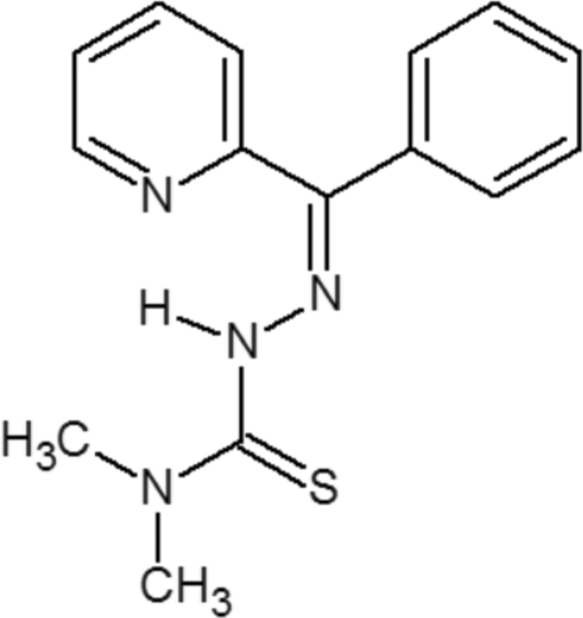


## Experimental

### 

#### Crystal data

C_15_H_16_N_4_S
*M* *_r_* = 284.39
Monoclinic, 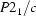

*a* = 10.011 (2) Å
*b* = 8.888 (2) Å
*c* = 16.256 (4) Å
β = 94.528 (3)°
*V* = 1441.9 (6) Å^3^
*Z* = 4
Mo *K*α radiation
μ = 0.22 mm^−1^
*T* = 153 K
0.32 × 0.28 × 0.22 mm

#### Data collection

Bruker P4 diffractometer
Absorption correction: multi-scan (*SADABS*; Bruker, 2004 ▶) *T*_min_ = 0.932, *T*_max_ = 0.953
14231 measured reflections
2828 independent reflections
2405 reflections with *I* > 2σ(*I*)
*R*_int_ = 0.031

#### Refinement

*R*[*F* ^2^ > 2σ(*F* ^2^)] = 0.039
*wR*(*F* ^2^) = 0.110
*S* = 1.06
2828 reflections
188 parameters
H atoms treated by a mixture of independent and constrained refinement
Δρ_max_ = 0.19 e Å^−3^
Δρ_min_ = −0.20 e Å^−3^

### 

Data collection: *SMART* (Bruker, 2004 ▶); cell refinement: *SAINT* (Bruker, 2004 ▶); data reduction: *SAINT*; program(s) used to solve structure: *SHELXS97* (Sheldrick, 2008 ▶); program(s) used to refine structure: *SHELXL97* (Sheldrick, 2008 ▶); molecular graphics: *SHELXTL* (Sheldrick, 2008 ▶) and *ORTEP-3* (Farrugia, 1997 ▶); software used to prepare material for publication: *SHELXL97* and *publCIF* (Westrip, 2010 ▶).

## Supplementary Material

Crystal structure: contains datablock(s) global, I. DOI: 10.1107/S1600536811045739/fj2463sup1.cif

Structure factors: contains datablock(s) I. DOI: 10.1107/S1600536811045739/fj2463Isup2.hkl

Supplementary material file. DOI: 10.1107/S1600536811045739/fj2463Isup3.cml

Additional supplementary materials:  crystallographic information; 3D view; checkCIF report

## Figures and Tables

**Table 1:** Hydrogen-bond geometry (Å, °) *Cg* is the centroid of the N1/C8–C12 ring.

*D*—H⋯*A*	*D*—H	H⋯*A*	*D*⋯*A*	*D*—H⋯*A*
N3—H3′⋯N1	0.837 (17)	1.869 (17)	2.602 (2)	145.4 (15)
C5—H5⋯*Cg*^i^	0.93	2.66	3.536 (2)	157

Symmetry code: (i) 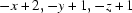.

**Fig. 1.**
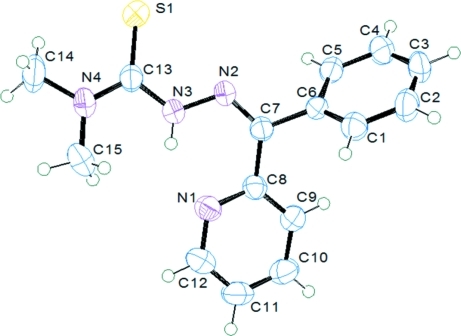
The molecular structure of the title compound with displacement ellipsoids are drawn at 50% probability level.

**Fig. 2.**
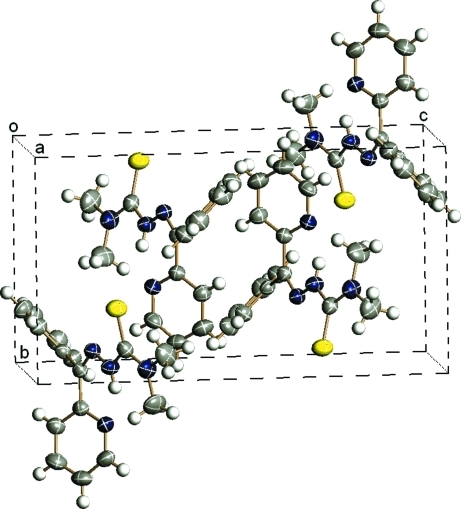
Packing diagram of the title compound, the unit cell is viewed down the a axis.

## supplementary crystallographic information

### Comment

A large number of studies have been devoted to the search for derivatives of hydrazinecarbothioamide, which have been used as drugs and have the ability to form complexes. The biological activity of these compounds depends on the parent aldehyde or ketone (Beraldo & Gambino, 2004). Derivatives of hydrazinecarbothioamide constitute an important group of multidentate ligands with potential binding sites available for a wide variety of metal ions. These thiourea derivatives find substantial applications in different facets of contemporary scientific research.

The title compound (Z)-2-N,N-dimethyl-2-[phenyl(pyridin-2-yl)methylidene]hydrazinecarbothioamide is found to exist in Z configuration. A perspective view of the molecular structure of the title compound, along with the atom-labeling scheme, is given in Fig. 1. The S1= C13–N3–N2 torsion-angle [14.4 (2)°] indicates that thionyl atom S1 is positioned cis to azomethane nitrogen atom N2. The hydrazinecarbothioamide moiety adopts an extended conjugation, with electron delocalization throughout the N4/C13/S1/N3/N2 group. The fact that the compound exists in the thione form is confirmed by the N3—N2, N4—C13 and C13=S1 bond distances. The C13=S1 bond distance is close to that expected for a C=S double bond of 1.60 Å (Huheey *et al.*, 1993). The N3—N2 bond distance is very close to the reported similar substituted hydrazinecarbothioamide (Joseph *et al.*, 2004). The resonance form involving pyridine ring would account for the shortening of the N—N distance through extensive electron delocalization.

The hydrazinecarbothioamide moiety, comprising atoms N3, C13, S1 and N4, is almost planar with the maximum deviation of 0.013 (2) Å for atom N4. The pyridyl ring and phenyl ring are not in the same plane and the pyridyl ring is twisted significantly from the hydrazinecarbothioamide plane, with a torsionl angle of -176.3 (2)°.

Two types of intramolecular (classical and non-classical) hydrogen bond interactions are found in this molecule. A classical hydrogen bonding interaction between the hydrogen attached to the N3 nitrogen and the N1 nitrogen with the D···A distance of 2.602 (2) Å and the non-classical hydrogen bonding interaction between one of the hydrogen atom attached to the C14 atom and the S1 atom of the molecule with a D···A distance of 3.030 (2) Å as described in Table 1.

Fig. 2 shows the packing diagram of the title compound. Packing of these molecules does not include any classical intermolecular hydrogen bonding interactions in its molecular array. However, it may be directed by the C—H···π interaction between the pyridine ring and the hydrogen attached at C5 carbon atom of the phenyl ring of the another molecule. There are four very weak π–π interactions present in this molecular array with the distances of 5.5874 (17), 4.8708 (15), 5.5455 (17) and 4.9165 (15) Å between the centroids of the corresponding rings involving interactions.

### Experimental

The title compound was prepared by adapting a reported procedure (Joseph *et al.*, 2006) by refluxing a mixture of methanolic solutions of 2-benzoylpyridine (11 mmol, 2.032 g) and N,N-dimethylhydrazinecarbothioamide (11 mmol, 1.320 g) for five hours after adding 5 drops of acetic acid. Yellow crystals were collected, washed with few drops of methanol and dried over P_4_O_10_ in vacuo. Single crystals of the title compound suitable for X-ray analysis were obtained by slow evaporation from its methanolic solution.

### Refinement

All H atoms on C were placed in calculated positions, guided by difference maps, with C—H bond distances 0.93–0.96 Å. H atoms were assigned as *U*_iso_=1.2Ueq (1.5 for Me). N3—H3' hydrogen was located from difference maps and restrained using *DFIX* instruction.

### Crystal data

**Table:**

C_15_H_16_N_4_S	*F*(000) = 600.0
*M**_r_* = 284.39	*D*_x_ = 1.310 Mg m^−^^3^
Monoclinic, *P*2_1_/*c*	Mo *K*α radiation, λ = 0.71073 Å
Hall symbol: -P 2ybc	Cell parameters from 8120 reflections
*a* = 10.011 (2) Å	θ = 2.0–26.0°
*b* = 8.888 (2) Å	µ = 0.22 mm^−^^1^
*c* = 16.256 (4) Å	*T* = 153 K
β = 94.528 (3)°	Block, yellow
*V* = 1441.9 (6) Å^3^	0.32 × 0.28 × 0.22 mm
*Z* = 4	

### Data collection

**Table:**

Bruker P4 diffractometer	2828 independent reflections
Radiation source: fine-focus sealed tube	2405 reflections with *I* > 2σ(*I*)
graphite	*R*_int_ = 0.031
Detector resolution: 8.33 pixels mm^-1^	θ_max_ = 26.0°, θ_min_ = 2.0°
ω scans	*h* = −12→12
Absorption correction: multi-scan (*SADABS*; Bruker, 2004)	*k* = −10→10
*T*_min_ = 0.932, *T*_max_ = 0.953	*l* = −20→20
14231 measured reflections	

### Refinement

**Table:**

Refinement on *F*^2^	Secondary atom site location: difference Fourier map
Least-squares matrix: full	Hydrogen site location: inferred from neighbouring sites
*R*[*F*^2^ > 2σ(*F*^2^)] = 0.039	H atoms treated by a mixture of independent and constrained refinement
*wR*(*F*^2^) = 0.110	*w* = 1/[σ^2^(*F*_o_^2^) + (0.0606*P*)^2^ + 0.2366*P*] where *P* = (*F*_o_^2^ + 2*F*_c_^2^)/3
*S* = 1.06	(Δ/σ)_max_ = 0.014
2828 reflections	Δρ_max_ = 0.19 e Å^−^^3^
188 parameters	Δρ_min_ = −0.20 e Å^−^^3^
0 restraints	Extinction correction: *SHELXL97* (Sheldrick, 2008), Fc^*^=kFc[1+0.001xFc^2^λ^3^/sin(2θ)]^-1/4^
Primary atom site location: structure-invariant direct methods	Extinction coefficient: 0.0104 (16)

### Special details

**Table:**

Geometry. All e.s.d.'s (except the e.s.d. in the dihedral angle between two l.s. planes) are estimated using the full covariance matrix. The cell e.s.d.'s are taken into account individually in the estimation of e.s.d.'s in distances, angles and torsion angles; correlations between e.s.d.'s in cell parameters are only used when they are defined by crystal symmetry. An approximate (isotropic) treatment of cell e.s.d.'s is used for estimating e.s.d.'s involving l.s. planes.
Refinement. Refinement of *F*^2^ against ALL reflections. The weighted *R*-factor *wR* and goodness of fit *S* are based on *F*^2^, conventional *R*-factors *R* are based on *F*, with *F* set to zero for negative *F*^2^. The threshold expression of *F*^2^ > σ(*F*^2^) is used only for calculating *R*-factors(gt) *etc*. and is not relevant to the choice of reflections for refinement. *R*-factors based on *F*^2^ are statistically about twice as large as those based on *F*, and *R*- factors based on ALL data will be even larger.

### Fractional atomic coordinates and isotropic or equivalent isotropic displacement parameters (Å^2^)

**Table:**

	*x*	*y*	*z*	*U*_iso_*/*U*_eq_	
S1	0.75460 (4)	0.15132 (5)	0.31975 (3)	0.05808 (18)	
N1	0.98755 (14)	0.68420 (14)	0.33757 (8)	0.0466 (3)	
N2	0.99018 (12)	0.35758 (13)	0.36663 (7)	0.0413 (3)	
N3	0.87523 (12)	0.42001 (16)	0.33015 (8)	0.0459 (3)	
N4	0.68025 (13)	0.41370 (17)	0.25113 (9)	0.0552 (4)	
C1	1.33165 (15)	0.42244 (19)	0.39734 (10)	0.0496 (4)	
H1	1.3333	0.4983	0.3580	0.059*	
C2	1.44999 (16)	0.3531 (2)	0.42682 (12)	0.0584 (5)	
H2	1.5307	0.3820	0.4069	0.070*	
C3	1.44848 (18)	0.2420 (2)	0.48527 (12)	0.0604 (5)	
H3	1.5280	0.1956	0.5048	0.072*	
C4	1.32983 (18)	0.1993 (2)	0.51494 (11)	0.0586 (5)	
H4	1.3291	0.1248	0.5551	0.070*	
C5	1.21090 (16)	0.26692 (18)	0.48536 (10)	0.0474 (4)	
H5	1.1305	0.2368	0.5053	0.057*	
C6	1.21111 (14)	0.37895 (16)	0.42634 (9)	0.0386 (3)	
C7	1.08395 (14)	0.45037 (16)	0.39224 (8)	0.0383 (3)	
C8	1.07742 (14)	0.61797 (16)	0.39196 (9)	0.0404 (3)	
C9	1.15590 (16)	0.70179 (18)	0.44910 (10)	0.0499 (4)	
H9	1.2173	0.6546	0.4865	0.060*	
C10	1.14187 (19)	0.85641 (19)	0.44978 (13)	0.0600 (5)	
H10	1.1928	0.9143	0.4882	0.072*	
C11	1.05114 (18)	0.92430 (19)	0.39262 (12)	0.0574 (4)	
H11	1.0408	1.0283	0.3912	0.069*	
C12	0.97729 (18)	0.83393 (18)	0.33834 (11)	0.0527 (4)	
H12	0.9165	0.8793	0.2998	0.063*	
C13	0.77074 (15)	0.33429 (18)	0.29902 (9)	0.0442 (4)	
C14	0.56134 (19)	0.3425 (3)	0.21167 (14)	0.0755 (6)	
H14A	0.5778	0.3140	0.1564	0.113*	
H14B	0.4877	0.4119	0.2101	0.113*	
H14C	0.5400	0.2547	0.2424	0.113*	
C15	0.6980 (2)	0.5728 (2)	0.23277 (14)	0.0770 (6)	
H15A	0.6945	0.6306	0.2824	0.115*	
H15B	0.6279	0.6051	0.1930	0.115*	
H15C	0.7833	0.5874	0.2108	0.115*	
H3'	0.8833 (15)	0.511 (2)	0.3188 (9)	0.043 (4)*	

### Atomic displacement parameters (Å^2^)

**Table:**

	*U*^11^	*U*^22^	*U*^33^	*U*^12^	*U*^13^	*U*^23^
S1	0.0506 (3)	0.0477 (3)	0.0740 (3)	−0.00580 (18)	−0.0075 (2)	−0.00052 (19)
N1	0.0516 (8)	0.0409 (7)	0.0469 (7)	0.0035 (6)	0.0016 (6)	0.0030 (5)
N2	0.0353 (6)	0.0410 (7)	0.0462 (7)	0.0030 (5)	−0.0045 (5)	0.0000 (5)
N3	0.0394 (7)	0.0388 (7)	0.0574 (8)	0.0035 (5)	−0.0087 (6)	0.0003 (6)
N4	0.0427 (7)	0.0588 (9)	0.0613 (8)	0.0069 (6)	−0.0143 (6)	−0.0006 (7)
C1	0.0441 (9)	0.0505 (9)	0.0542 (9)	0.0001 (7)	0.0042 (7)	0.0044 (7)
C2	0.0347 (8)	0.0697 (12)	0.0705 (11)	0.0005 (8)	0.0022 (8)	−0.0032 (9)
C3	0.0425 (9)	0.0639 (11)	0.0711 (11)	0.0098 (8)	−0.0182 (8)	−0.0052 (9)
C4	0.0562 (10)	0.0552 (10)	0.0611 (10)	0.0010 (8)	−0.0157 (8)	0.0113 (8)
C5	0.0418 (8)	0.0478 (9)	0.0513 (9)	−0.0055 (7)	−0.0045 (7)	0.0039 (7)
C6	0.0363 (7)	0.0361 (7)	0.0421 (8)	0.0002 (6)	−0.0042 (6)	−0.0050 (6)
C7	0.0372 (7)	0.0394 (7)	0.0380 (7)	0.0016 (6)	0.0014 (6)	−0.0007 (6)
C8	0.0373 (7)	0.0400 (8)	0.0442 (8)	0.0019 (6)	0.0058 (6)	0.0004 (6)
C9	0.0448 (9)	0.0463 (9)	0.0577 (9)	−0.0007 (7)	−0.0016 (7)	−0.0048 (7)
C10	0.0582 (11)	0.0457 (10)	0.0756 (12)	−0.0065 (8)	0.0024 (9)	−0.0123 (8)
C11	0.0634 (11)	0.0360 (8)	0.0745 (11)	−0.0007 (8)	0.0162 (9)	−0.0006 (8)
C12	0.0595 (10)	0.0438 (9)	0.0553 (9)	0.0074 (7)	0.0069 (8)	0.0092 (7)
C13	0.0373 (8)	0.0499 (9)	0.0444 (8)	0.0043 (6)	−0.0021 (6)	−0.0052 (6)
C14	0.0502 (11)	0.0935 (16)	0.0781 (13)	0.0042 (10)	−0.0250 (10)	−0.0092 (11)
C15	0.0699 (13)	0.0660 (13)	0.0902 (15)	0.0183 (10)	−0.0245 (11)	0.0121 (11)

### Geometric parameters (Å, °)

**Table:**

S1—C13	1.6712 (17)	C5—C6	1.383 (2)
N1—C12	1.335 (2)	C5—H5	0.9300
N1—C8	1.3461 (19)	C6—C7	1.4898 (19)
N2—C7	1.2934 (18)	C7—C8	1.491 (2)
N2—N3	1.3697 (17)	C8—C9	1.386 (2)
N3—C13	1.3591 (19)	C9—C10	1.381 (2)
N3—H3'	0.837 (17)	C9—H9	0.9300
N4—C13	1.3466 (19)	C10—C11	1.385 (3)
N4—C14	1.452 (2)	C10—H10	0.9300
N4—C15	1.459 (3)	C11—C12	1.366 (2)
C1—C6	1.385 (2)	C11—H11	0.9300
C1—C2	1.387 (2)	C12—H12	0.9300
C1—H1	0.9300	C14—H14A	0.9600
C2—C3	1.371 (3)	C14—H14B	0.9600
C2—H2	0.9300	C14—H14C	0.9600
C3—C4	1.370 (3)	C15—H15A	0.9600
C3—H3	0.9300	C15—H15B	0.9600
C4—C5	1.385 (2)	C15—H15C	0.9600
C4—H4	0.9300		
			
C12—N1—C8	118.54 (14)	N1—C8—C7	117.75 (13)
C7—N2—N3	116.40 (12)	C9—C8—C7	120.85 (13)
C13—N3—N2	121.99 (13)	C10—C9—C8	119.18 (16)
C13—N3—H3'	123.1 (11)	C10—C9—H9	120.4
N2—N3—H3'	113.3 (11)	C8—C9—H9	120.4
C13—N4—C14	121.18 (15)	C9—C10—C11	119.31 (16)
C13—N4—C15	122.61 (14)	C9—C10—H10	120.3
C14—N4—C15	116.16 (14)	C11—C10—H10	120.3
C6—C1—C2	120.13 (16)	C12—C11—C10	118.02 (15)
C6—C1—H1	119.9	C12—C11—H11	121.0
C2—C1—H1	119.9	C10—C11—H11	121.0
C3—C2—C1	120.23 (16)	N1—C12—C11	123.63 (16)
C3—C2—H2	119.9	N1—C12—H12	118.2
C1—C2—H2	119.9	C11—C12—H12	118.2
C4—C3—C2	120.04 (16)	N4—C13—N3	112.61 (14)
C4—C3—H3	120.0	N4—C13—S1	123.72 (12)
C2—C3—H3	120.0	N3—C13—S1	123.65 (11)
C3—C4—C5	120.19 (16)	N4—C14—H14A	109.5
C3—C4—H4	119.9	N4—C14—H14B	109.5
C5—C4—H4	119.9	H14A—C14—H14B	109.5
C6—C5—C4	120.31 (15)	N4—C14—H14C	109.5
C6—C5—H5	119.8	H14A—C14—H14C	109.5
C4—C5—H5	119.8	H14B—C14—H14C	109.5
C5—C6—C1	119.09 (14)	N4—C15—H15A	109.5
C5—C6—C7	121.12 (13)	N4—C15—H15B	109.5
C1—C6—C7	119.77 (13)	H15A—C15—H15B	109.5
N2—C7—C6	115.17 (12)	N4—C15—H15C	109.5
N2—C7—C8	127.26 (13)	H15A—C15—H15C	109.5
C6—C7—C8	117.56 (12)	H15B—C15—H15C	109.5
N1—C8—C9	121.30 (14)		
			
C7—N2—N3—C13	178.44 (14)	N2—C7—C8—N1	−23.7 (2)
C6—C1—C2—C3	−0.5 (3)	C6—C7—C8—N1	157.05 (13)
C1—C2—C3—C4	−0.2 (3)	N2—C7—C8—C9	152.63 (15)
C2—C3—C4—C5	0.8 (3)	C6—C7—C8—C9	−26.6 (2)
C3—C4—C5—C6	−0.7 (3)	N1—C8—C9—C10	−0.4 (2)
C4—C5—C6—C1	0.0 (2)	C7—C8—C9—C10	−176.54 (16)
C4—C5—C6—C7	178.48 (14)	C8—C9—C10—C11	−0.9 (3)
C2—C1—C6—C5	0.6 (2)	C9—C10—C11—C12	1.0 (3)
C2—C1—C6—C7	−177.91 (14)	C8—N1—C12—C11	−1.5 (3)
N3—N2—C7—C6	−175.96 (12)	C10—C11—C12—N1	0.2 (3)
N3—N2—C7—C8	4.8 (2)	C14—N4—C13—N3	179.72 (16)
C5—C6—C7—N2	−50.15 (19)	C15—N4—C13—N3	2.5 (2)
C1—C6—C7—N2	128.32 (15)	C14—N4—C13—S1	−1.7 (2)
C5—C6—C7—C8	129.21 (15)	C15—N4—C13—S1	−178.84 (15)
C1—C6—C7—C8	−52.32 (19)	N2—N3—C13—N4	−166.98 (14)
C12—N1—C8—C9	1.5 (2)	N2—N3—C13—S1	14.4 (2)
C12—N1—C8—C7	177.81 (14)		

### Hydrogen-bond geometry (Å, °)

**Table:**

Cg is the centroid of the N1/C8–C12 ring.

**Table:**

*D*—H···*A*	*D*—H	H···*A*	*D*···*A*	*D*—H···*A*
N3—H3'···N1	0.837 (17)	1.869 (17)	2.602 (2)	145.4 (15)
C14—H14C···S1	0.96	2.57	3.030 (2)	109.
C5—H5···Cg^i^	0.93	2.66	3.536 (2)	157

Symmetry codes: (i) −*x*+2, −*y*+1, −*z*+1.
